# RESEARCH, (UN)INTERRUPTED? EFFECTS OF THE PANDEMIC ON A 1 YEAR FOLLOW-UP TO AN ONLINE RCT

**DOI:** 10.1093/geroni/igac059.1784

**Published:** 2022-12-20

**Authors:** Alexandra Jeanblanc, Carol Musil, Christopher Burant, Jaclene Zausniewski

**Affiliations:** Case Western Reserve University, Cleveland, Ohio, United States; Case Western Reserve University, Cleveland, Ohio, United States; Case Western Reserve University, Parma, Ohio, United States; Case Western Reserve University, Cleveland, Ohio, United States

## Abstract

The pandemic profoundly disrupted research, largely due to the interruption of data collection. Our online-only behavioral RCT, evaluating two methods of stress-reduction for grandmothers with co-resident grandchildren, completed the originally planned 4 data collection points prior to the pandemic. When it began, we were in the process of collecting a 5th questionnaire (Q5) from a sample of 152. Our pre-existing work from home policies allowed us to continue data collection uninterrupted.Of 152 potential grandmothers, 51 (58 eligible) grandmothers completed Q5 prior to the pandemic and 62 (94 eligible) completed it after it began, (total N=113, 74.43% response rate. We compared Q5 responses pre/post pandemic, finding no significant differences on resilience, mindfulness/decentering, subjective support, instrumental support, or the friendship scale. Post-pandemic participants had significantly lower scores on the social resourcefulness subscale (t=-1.723, p=.044), but not personal or overall resourcefulness. They reported more depressive symptoms for the CES-D positive affect and depressed affect subscales (t=1.997, p=.048; t=1.673, p=.049). The post-pandemic sample reported worse self-rated health over the last year (t=2.753, p=.003) and worse overall health (t=1.781, t=.039).The higher levels of depressive symptoms, worse self-reported health, and lower social resourcefulness aligns with pandemic-related changes observed by other studies. We did not need to adapt our research procedures for the pandemic and, due to the lower post-pandemic response rate, our subsamples were of relatively equal size. Consequently, it is likely that the differences we observe here are real and reflect the negative effect of the pandemic on individual health and psycho-social well-being.

